# ^18^FDG-PET-CT identifies histopathological non-responders after neoadjuvant chemotherapy in locally advanced gastric and cardia cancer: cohort study

**DOI:** 10.1186/s12885-018-4477-4

**Published:** 2018-05-09

**Authors:** Paul M. Schneider, Dilmurodjon Eshmuminov, Tamara Rordorf, Diana Vetter, Patrick Veit-Haibach, Achim Weber, Peter Bauerfeind, Panagiotis Samaras, Kuno Lehmann

**Affiliations:** 1 Center for Visceral, Thoracic and specialized Tumor Surgery, Hirslanden Medical Center, Witellikerstrasse 40, CH-8032 Zurich, Switzerland; 2 Department of Visceral and Transplantation Surgery, University Hospital Zurich, Zurich, Switzerland; 3 Department of Oncology, University Hospital Zurich, Zurich, Switzerland; 4 Department of Nuclear Medicine, University Hospital Zurich, Zurich, Switzerland; 5 Institute of Clinical Pathology, University Hospital Zurich, Zurich, Switzerland; 6 Department of Gastroenterology, University Hospital Zurich, Zurich, Switzerland; 7 Oncology Centre, Hirslanden Medical Center, Zurich, Switzerland

**Keywords:** Histopathologic regression, PET-CT, Gastric cancer, AEG

## Abstract

**Background:**

Pathologic response to neoadjuvant chemotherapy (neoCTX) is a prognostic factor in many cancer types, and early prediction would help to modify treatment. In patients with gastric and esophagogastric junction (AEG) cancer, the accuracy of FDG PET-CT to predict early pathologic response after neoadjuvant chemotherapy (neoCTX) is currently not known.

**Methods:**

From a consecutive cohort of 72 patients, 44 patients with resectable, locally-advanced gastric cancer or AEG Siewert type II and III received neoCTX after primary staging with endoscopic ultrasound, PET-CT and laparoscopy. Overall, 14 patients did not show FDG uptake, and the remaining 30 were restaged by PET-CT 14 days after the first cycle of neoCTX. Metabolic response was defined as decrease of tumor standardized uptake value (SUV) by ≥35%. Major pathologic regression was defined as less than 10% residual tumor cells.

**Results:**

Metabolic response after neoCTX was detected in 20/30 (66.7%), and non-response in 10/30 (33.3%) patients. Among metabolic responders, *n* = 10 (50%) showed major and n = 10 (50%) minor pathologic regression. In non-responders, *n* = 9 (90%) had minor and 1 (10%) a major pathologic regression. This resulted in a sensitivity of 90.9%, specificity 47.3%, positive predictive value 50%, negative predictive value 90% and accuracy of 63.3%.

**Conclusion:**

Response PET-CT after the first cycle of neoCTX does not accurately predict overall pathologic response. However, PET-CT reliably detects non-responders, and identifies patients who should either immediately proceed to resection or receive a modified multimodality therapy.

**Trial registration:**

The trial was registered and approved by local ethics committee PB_2016–00769.

## Background

Cancer of the stomach (GC) and the distal esophagus and esophagogastric junction (AEG) is a substantial global health problem with around 1 million new cases and 750,000 deaths per year, accounting for estimated 10% of all cancer-related deaths [1, 2]. In Europe and North America, the overall 5-year survival for GC is approximately 25% [3], while superior outcomes with 5-year survival rates of approximately 60% are reported in East Asia [4].

The optimal medical treatment for advanced GC and AEG is still a source of debate, but after the publication of the randomized “MAGIC Trial” and “ACCORD Trial”, neoadjuvant chemotherapy has become first choice for the treatment of locally advanced GC and AEG, and reported improved 5-year survival rates of 36 and 38% respectively [5, 6]. This situation is similar for esophageal and cardia cancers where the recently published randomized “CROSS Trial”, using neoadjuvant chemoradiation, reported 3-year survival rates of 59% [7].

Objective assessment of the treatment effect after neoadjuvant treatment is only possible by histopathology in the resected specimens. In patients with AEG and GC, the presence of < 10% of vital residual tumor cells is considered as a major pathologic response, and is associated with a significant survival benefit [8–10]. The difficulty is to identify patients who do not respond or progress under neoadjuvant treatment. Those patients may not profit from neoadjuvant treatment, still suffer from adverse events, and finally risk tumor progression.

In the setting of esophageal cancer, measurement of early changes in tumor glucose uptake by use of 18-fluorodeoxyglucose-PET (PET) and later PET-CT yielded promising results for predicting response following neoadjuvant chemotherapy [11]. Metabolic tumor activity can be quantified by the standardized uptake value (SUV), and it was shown that a drop of ≥35% measured after 2 weeks of induction chemotherapy was an accurate cut-off value to predict response [12]. This cut-off at ≥35% was prospectively studied in the MUNICON-I trial including 119 patients with AEG I and II [13]. Metabolic response evaluation by PET-CT accurately identified all non-responding tumors within two weeks of treatment. In addition, there was a significant survival difference between metabolic responders and non-responders.

No data are currently available for early metabolic response evaluation by PET-CT in patients with AEG III and GC, and the potential benefit is therefore unclear. We studied patients with AEG II/III and GC in a cohort of patients using the criteria for early metabolic response from the MUNICON-I trial to evaluate the accuracy and feasibility of metabolic response evaluation by early PET-CT following neoadjuvant CTX.

## Methods

A retrospective cohort of 72 consecutive patients with biopsy proven GC or AEG Siewert type II-III [14] was included in this study. All patients underwent routine staging, including, laboratory tests, upper GI-endoscopy with endoscopic ultrasound (EUS) and ^18^FDG PET-CT as reported previously [15]. Patients with stage cT2N+ or cT3–4, Nx by EUS and PET-CT (UICC TNM Classification, 7th edition, [16] underwent diagnostic laparoscopy to exclude occult peritoneal carcinomatosis prior to neoCTX. All patients were discussed in a multidisciplinary specialized tumor board prior to treatment initiation. The study was approved by the local ethics committee (PB 2016–00769).

### Imaging by ^18^FDG positron emission tomography-CT

A baseline PET-CT was performed as part of the staging procedure and an early response PET-CT, 14 days after the first cycle of neoadjuvant CTX. Imaging was performed on an in-line system (Discovery RX or Discovery VCT; GE Healthcare). These systems integrate a state-of-the art full ring PET scanner with a multi-slice helical CT (LightSpeed 16 or VCT 64 slice; GE Healthcare) allowing for acquisition of co-registered CT and PET images in one session. Patients fasted for at least 4 h before scanning, which started 50–60 min after the injection of a standard dose of 340–370 MBq 18F-FDG. A low dose CT (80 mA, 140 kV, 0.5-s tube rotation, 4.25-mm section thickness, 867-mm scan length, and 22.5-s data acquisition time) was performed first. Immediately after CT, the PET emission scan was acquired, with 2 min emission time per cradle position (total PET-CT acquisition time 12–16 min). PET images were reconstructed using a standard 3-dimensional iterative algorithm (ordered-subset expectation maximization). Image reading was done on screen using a commercially available software package (Advantage workstation, version 4.4; GE Healthcare). For quantitative measurement, a circular region of interest was placed over the tumor in the slice with maximum [18F]-FDG uptake in the baseline scan. A tumor was defined as negative or non-avid when there was no measurable activity over background [18F]-FDG uptake in the tumor area defined by endoscopy and/or demonstrated as mass in the integrated multi-slice CT. In the second PET scan, the region of interest was placed according to the baseline study using the surrounding anatomical landmarks. Patients with a decrease of ≥35% SUV were classified as metabolic responders (12,13).

### Neoadjuvant chemotherapy

Two neoadjuvant chemotherapy regimens were applied, either 3 cycles ECF according the “MAGIC” regimen [5], or 3 cycles of FLOT, consisting of biweekly oxaliplatin 85 mg/m2, day1; docetaxel 50 mg/m2, day2; and continuous infusion 5-FU 2600 mg/m2 days 1–2 [17, 18]. Adverse events were reported according to the National Cancer Institute Criteria, version 3.0.

### Surgery

Standardized resections were performed including subtotal (80%) gastrectomy for distal GC, gastrectomy for middle or proximal third GC and transhiatal extended gastrectomy for AEG Siewert type II-III tumors [19]. Systematic D2-lymphadenectomy (LAD) was routinely performed [20], and additionally LAD of the lower mediastinum for AEG types II/III. In few selected patients, para-aortic lymph node dissection (D3-LAD, [20]) was performed. Complications were recorded using the Clavien-Dindo classification [21].

### Pathology

Pathologic tumor regression was evaluated using a published validated scoring system [8]. Patients with less than 10% residual tumor cells were classified as responders. All other patients were classified as non-responders. All specimens were reviewed by an experienced gastrointestinal pathologist (AW).

### Follow-up

Patients were followed clinically and by contrast CT (local tumor recurrence, lymph node metastases, systemic metastases including peritoneal carcinomatosis) at 4-month intervals during the first year after surgery and at 6-month intervals thereafter, and endoscopy at 6 months and yearly thereafter. Survival was calculated from the day of study inclusion.

### Statistical analysis

Differences in proportions were analyzed using Fisher’s exact test. Inter-individual comparisons of quantitative data were done by use of a Wilcoxon signed rank test. Survival was estimated according to Kaplan-Meier. Statistical comparisons between different groups of patients were done with a log-rank test. All tests were two-sided and done at the 5% level of significance with the use of SPSS for Windows, version 11.50 (SPSS Inc., Chicago, IL, USA).

## Results

### Study population

From October 1, 2008 to October 31, 2013, 72 consecutive patients with resectable, locally-advanced GC or AEG II/III were included. Among them, 28 patients were finally not eligible for neoadjuvant chemotherapy due to severe comorbidities, patient’s decision, or peritoneal carcinomatosis at diagnostic laparoscopy. In 44 patients planned for neoCTX, 14 (32%) did not show FDG uptake and could therefore not be further evaluated. The remaining 30 patients (68%) underwent neoCTX and were restaged by PET-CT 14 days after beginning neoCTX. Patient characteristics are shown in Table 1.

**Table 1:** Patient characteristics

Parameter	Median (range)	*n* = 30	%
Age (years)	57.4 (36.9–78.9)		
Gender			
male		22	73
emale		8	27
Body Mass Index (kg/m^2^)	23.2 (16.7–30.8)		
Charlson-comorbidity-Index			
2		23	77
3–5		4	13
> 6		3	10
ECOG score			
0		22	73
1		8	27
Localization			
AEG Siewert Type II		12	40
AEG Siewert Type III		10	34
Gastric cancer		8	26
Grading			
G2		14	47
G3		16	53
Laurén’s classification			
Intestinal		24	80
Mixed		3	10
Diffuse		3	10
uT-category			
uT2		4	13
uT3		22	74
uT4		4	13
cN-category			
cN0		3	10
cN+		27	90
cM-category			
cM0		24	80
cM1		6	20

Clinical TNM staging is based on EUS (uT) and/or CT or PET-CT (cN)

### Chemotherapy

Nine patients (30%) received neoCTX with ECF [5], and 21 (70%) received FLOT [17, 18]. 8/9 patients pretreated with ECF received the planned 3 cycles and one patient refused the third cycle because of side effects. 20/21 (95%) patients with FLOT received 3 full cycles and 1 patient received 2 cycles due to severe bone marrow depression.

### Surgery and perioperative complications

All but one patient proceeded to surgical resection within 3 weeks, while it had to be postponed to week 5 due to bone marrow depression. Radical lymphadenectomy was performed in all patients with a high median number of resected lymph nodes of 43 (range 23–113). Perioperative morbidity according to the Clavien-Dindo classification was observed in 50% of patients, including major complications grade IIIb in 4 (13%) and grade IV in 1 (4%) patient. There was no in-hospital or 90-day mortality. Surgical details are summarized in Table 2.

**Table 2:** Surgical characteristics

Parameter	*n* = 30	%
Type of resection		
Subtotal gastrectomy	2	7
Gastrectomy	2	7
Extended gastrectomy	2	7
Transhiatal extended gastrectomy^a^	22	72
Esophagectomy	2	7
Type of lymphadenectomy		
D2-lymphadenectomy	4	13
D2+ lower mediastinum	20	13
D3-paraaortic ± lower mediastinum	4	67
2-field (abdominal and extended mediastinal)	2	7
R-category		
R0	25	83
R1/R2	5	17
Postoperative complications (Clavien-Dindo)		
none	15	50
Grade I	3	10
Grade II	7	23
Grade IIIa	0	0
Grade IIIb	4	13
Grade IVa	1	4
Grade IVb	0	0
Grade V	0	0

^a^Splenectomy: *n* = 1

### Pathology

Most patients had locally-advanced ypT3–4 tumors (83%), and positive lymph nodes (67%). Advanced N-categories ypN2–3 were detected in 50% of resected specimens. Major pathologic regression occurred in 11/30 (36.7%) tumors with only 2/30 (7%) showing a pathologic complete remission. Major pathologic response in R0-resected patients was significantly associated with improved median survival rates (not reached) compared to minor response (28.2 months; 95% CI 16.7–39.7 months) by log-rank test (*p* = 0.04). Results are summarized in Table 3.

**Table 3:** Histopathology after neoadjuvant chemotherapy

Parameter	Median (range)	*n* = 30	%
ypT-category			
ypT0		2	7
ypT1		2	7
ypT2		1	3
ypT3		21	70
ypT4a		3	10
ypT4b		1	3
Number of removed lymph nodes	43 (21–113)		
ypN-category			
ypN0		10	33
ypN1		5	17
ypN2		7	23
ypN3		8	27
ypN3 (AEG)		3	10
ypN3a (GC)		4	14
ypN3b (GC)		1	3
Tumor regression grading			
Complete regression (Ia)		2	7
< 10% residual tumor (Ib)		9	30
≥ 10% and < 50% residual tumor (II)		6	20
≥ 50% residual tumor (III)		8	27
no regression (IV)		5	16
Modified tumor regression grading			
Minor (grade II - IV, ≥ 10% residual tumor)		19	63
Major (grade Ia - Ib, < 10% residual tumor)		11	37

### Metabolic response

In 30 patients with PET positive primary tumors, median SUV significantly decreased from 10.4 (range 4.0–29.8) to 5.0 (range 0–25.2) 14 days after the first cycle neoCTX (*p* < 0.0001). Metabolic response was observed in 20 (66.7%), and no response in 10 (33.3%) patients. Prediction of pathologic response by metabolic response on PET-CT resulted in a sensitivity of 90.9% (95%-CI: 57.1–99.5%), specificity 47.3% (95%-CI: 25.2–70.5%), positive predictive value (PPV) 50% (95%-CI: 27.8–72.1%), negative predictive value (NPV) 90% (95%-CI: 54.1–99.4%) and overall accuracy of 63.3% (95% CI: 38.5–78.6%). Although the overall accuracy is low, the NPV is high with a correct identification in 9/10 true non-responding tumors.

### Metabolic response and prognosis

**Fig. 1:**
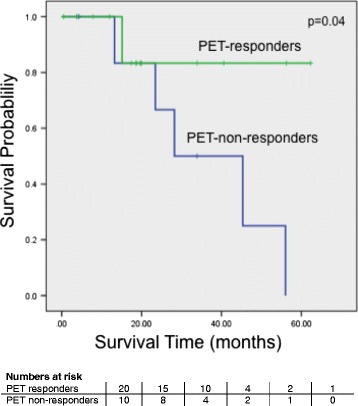
Overall survival according response PET-CT Overall survival estimates of *n* = 30 patients according to Kaplan-Meier curves based on their response PET-CT after two cycles of neoCTX. Numbers at risk are shown in 12 month intervals

Median follow-up was 22.4 months (range 3.2–61.8) for surviving patients. Median overall survival was significantly better for metabolic responders than for non-responders (median survival not reached and 28.2 months, 95%-CI: 7.2–10.7 months, respectively, log-rank *p* = 0.04). Survival curves are shown in Fig. 1.

## Discussion

This cohort study in patients with locally advanced AEG II/III and GC shows that metabolic response two weeks after starting neoadjuvant therapy evaluation by FDG-PET-CT, using the validated threshold for metabolic responders from the MUNICON-I intervention trial (13) of ≥35% reduction of SUV does not accurately predict overall pathologic response. It does however identify a subgroup of patients that does not respond to neoCTX with a specificity of 90%. In this cohort, this subgroup compromised 14% (10/72) of the study population with an inferior prognosis compared to PET responders.

The results of our study compare well to the results of the MUNICON-1 trial in patients with esophageal cancer, where 110 patients with AEG I/II were evaluated by PET-CT [13]. The authors claim that responders can be identified by early metabolic imaging, however, 42% of the 50 PET responders showed a minor regression like the 50% observed in our study. The NPV for the metabolic response was 100% in the MUNICON trial, which is comparable to the 90% in our study and the 10% difference is likely due to the low patient numbers and different tumor entities. We therefore conclude that metabolic response evaluation by PET-CT does not accurately predict overall response but identifies non-responders in both trials. We also confirmed that PET-responders have a better prognosis than non-responders (*p* = 0.04) with remarkably close survival rates (median survival was not reached in PET-responders in both trials and 26 months and 28.2 months respectively for PET non-responders). In the MUNICON-I intervention trial, chemotherapy was discontinued in metabolic non-responders, thereby saving time, and reducing side-effects and costs without compromising the outcome.

Less data is available for patients with gastric cancer. Vallböhmer et al. [22] found no predictive value for the FDG uptake in 40 gastric cancer patients. Ott and colleagues [23] prospectively studied 49 GC patients including Siewert type III tumors with a metabolic response (SUV reduction ≥35%) by PET. Overall, 23/49 (47%) patients had non-intestinal type cancers and 38/49 (78%) were in the proximal third. Metabolic response correctly predicted histopathologic regression in 11/16 responding and 27/33 non-responding tumors. This resulted in a sensitivity and specificity of 69 and 82%. PPV and NPV were 65 and 84% and overall accuracy was 78%. Median survival of metabolic responders was not reached, and significantly (*p* = 0.037) better than for non-responders (24.1 months). Again, remarkably similar results were obtained in our study. The lower NPV is likely attributable to the higher proportion of non-intestinal tumors (46.9%) compared to our study (20%) and the significantly lower baseline SUV obtained in non-intestinal-type tumors.

In contrast to patients receiving chemotherapy alone, early metabolic response evaluation by PET-CT was not successful in patients receiving neoadjuvant chemoradiation for esophageal and esophagogastric junction cancers. [24–26]. Technically, a higher cut-off value might be better to predict histopathological response. Indeed, a previous study from the Munich group [12] showed that a 45% or more decrease in SUV would result in higher specificity for histological response (86% versus 75%) but most important, in a lower NPV. This was also true in our study with a cut-off at 50% (data not shown). The FDG uptake is not uniform among the subgroups. Esophageal tumors, AEG I, show about 100% FDG uptake, much higher than AEG III or GC, or diffuse type cancer [15]. Therefore, the use of PET-CT is not uniformly recommended, particularly in non-intestinal gastric cancer including signet ring cell cancer [23, 27–29].

Distal gastric cancer with low differentiation grades, including diffuse types are less likely to achieve major tumor regression after chemotherapy [30]. A risk score was evaluated in 410 patients receiving neoCTX for GC. Well-differentiated tumor grading, intestinal tumor type histology and tumor localization in the middle third of the stomach were identified as the significant positive predictive factors for histopathologic response and prognosis. A prognostic index could be created based on tumor localization, grading and type according to Lauren classification that identified 3 risk groups (low, intermediate and high) with significantly different clinical and histopathological response rates and overall survival times. [31]. Several molecular markers have been investigated in view of characterizing tumor entities and predicting tumor response and prognosis following neoadjuvant treatment [32, 33], since a variety of novel targeted therapeutic approaches are introduced in cancer treatment [34]. In HER2-positive advanced GC and AEG, the international phase III trastuzumab for GC (ToGA) study showed a significant improvement in the median overall survival of patients upon the addition of trastuzumab to cisplatin and fluoropyrimidine backbone therapy [35]. In the MUNICON-II trial, salvage neoadjuvant radiochemotherapy in metabolic non-responders lead to local remissions in a considerable number of patients but was not able to change the clinical course [36]. Altogether, metabolic non-responders may profit from a therapeutic switch to these novel approaches.

Our study clearly has its limitations, mainly attributable to the small study population which does not allow more sophisticated statistical analysis including multivariate testing, and all test results must therefore be interpreted with caution. Despite these limitations, our results are remarkably close to the results of two published comparable studies, the MUNICON PET-CT trial in Siewert type I/II tumors [13] and the PET-study in gastric cancer including Siewert type III tumors [23] using early metabolic response evaluation. Furher research could also include assessing survival outcomes in patients classified as non-responders by PET-CT with subsequent discontinuation of neoadjuvant chemotherapy and comparing them with those remaining on treatment.

## Conclusions

In conclusion, our study in patients with AEG and GC adds further evidence that response PET-CT reliably detects metabolic non-responders and can therefore identify patients who should either immediately proceed to resection or receive a modified multimodality therapy. PET-CT-guided neoadjuvant chemotherapy appears feasible in patients with AEG and GC but important issues remain to be addressed in future trials especially standardization for metabolic imaging as planned by the EORTC GI Group and NEOPEC Trial Group [37, 38].

## Abbreviations

AEG: Adenocarcinoma of the esophagogastric junction
CT: Computed tomography
EUS: Endoscopic ultrasound
GC: Gastric cancer
MDCT: Multidetector spiral computed tomography
neoCTX: neoadjuvant chemotherapy
PET: 18-fluorodeoxyglucose (^18^FDG) positron emission tomography
PET-CT: Combined positron emission tomography and computed tomography
